# Distribution and associated factors of keratometry and corneal astigmatism in an elderly population

**DOI:** 10.1038/s41598-024-70850-4

**Published:** 2024-08-26

**Authors:** Hassan Hashemi, Mohamadreza Aghamirsalim, Alireza Hashemi, Mehdi Khabazkhoob

**Affiliations:** 1 Noor Ophthalmology Research Center, Noor Eye Hospital, Tehran, Iran; 2 Translational Ophthalmology Research Center, Tehran University of Medical Sciences, Tehran, Iran; 3 Noor Research Center for Ophthalmic Epidemiology, Noor Eye Hospital, Tehran, Iran; 4 Department of Medical Surgical Nursing, School of Nursing and Midwifery, Shahid Beheshti University of Medical Sciences, Tehran, Iran

**Keywords:** Epidemiology, Outcomes research

## Abstract

To determine the distribution of keratometry and corneal astigmatism (CA) and their association with demographic factors, systemic parameters, anthropometric measures, ocular biometric indices, and refractive errors in people aged 60 years and above. In this cross-sectional study, 160 clusters were randomly selected from Tehran city (Iran) using the multi-stage cluster sampling method. All participants underwent optometric examinations including testing uncorrected and best-corrected distance visual acuity, non-cycloplegic autorefraction, and subjective refraction. Pentacam imaging for all participants was carried out using Pentacam AXL. Keratometry and CA were reported based on Pentacam’s data. The average, standard deviation (SD) and 95% confidence interval (CI) of flat keratometry (Kf), steep keratometry (Ks), mean keratometry (mean K), and CA were 44.02 ± 1.58 D (95% CI 43.94–44.1), 44.86 ± 1.67 D (95% CI 44.78–44.94), 44.44 ± 1.58 D (95% CI 44.36–44.52), and 0.84 ± 0.74 D (95% CI 0.81–0.87), respectively. The 95% and 99% percentiles of mean K were 47.1 and 48.6 D, respectively. According to the multiple generalized estimating equation model, the mean K was significantly higher in males, in myopes, and in those with higher systolic blood pressure. Moreover, the mean K was inversely related to the axial length, height, anterior chamber depth (ACD), corneal diameter, and central corneal thickness (CCT). The prevalence of various types of CA based on a cut-off > 0.50 D was as follows; with-the-rule: 32.5% (95% CI 30.6–34.4), against-the-rule: 18.2% (95% CI 16.7–19.7), and oblique: 10.0% (95% CI 9.1–11.0). The present study investigated the normal distribution of keratometry and CA in individuals ≥ 60 years, and results can be used in clinical matters, especially in intraocular lens power calculation. Sex, systolic blood pressure, height, and some biometric components such as ACD, corneal diameter, and CCT were significantly related to keratometry and should be considered.

## Introduction

Knowledge of ocular biometric components is important for diagnostic and therapeutic purposes, as well as for ocular surgeries^1–5^. In some ocular surgeries, information on some biometric components is essential. Axial length (AL)^6^, corneal power^7^, corneal thickness^8^, and anterior chamber depth (ACD)^9^ are perhaps the most widely used ocular biometric components required in most surgical procedures^10^. Among these, corneal power or keratometry is a key parameter to consider from a young age when managing keratoconus (KCN) to old age when calculating the intraocular lens (IOL) power before cataract surgery^11–14^.

Advancing knowledge and practical experience have led to cataract surgery becoming a refractive surgery and a significant increase in patients' expectations for optimal uncorrected visual acuity. Accurate calculation of the IOL power is an important step in achieving patient satisfaction with the cataract surgery outcome. Keratometry is considered an important element in the newer generation IOL power calculation formulas including artificial intelligence (AI)-based formulas^15^. Although keratometry was used in older formulas, the newer IOL power formulas even consider the curvature of the posterior corneal surface^16^. Keratometry plays a vital role in deciding whether to correct corneal astigmatism (CA) with toric lenses or improve near vision with multi-focal or accommodative IOLs during cataract surgery^17,18^.

The eye structure undergoes physiological and pathological changes with aging that can be difficult to differentiate^19^. Changes in corneal curvature and power are associated with the emmetropization process from childhood and these changes lead to the steepening of the corneal curvature and transition of astigmatism from with-the-rule (WTR) to against-the-rule (ATR) in older adults^20,21^. Studying the pattern of changes with age can help understanding the nature of changes and taking appropriate action, when necessary.

Knowing normative keratometry values in various populations and ethnicities helps to create new IOL power calculation formulas by AI and analyze corneal changes in those communities. This information can even help identify different KCN patterns. In addition to race and ethnicity, keratometry is also affected by other factors^20^.

Although many devices are capable of measuring keratometry, the use of Sheimpflug imaging technology by the Pentacam has provided practitioners with more accurate keratometry data in recent years^22^. In addition to calculating the corneal power based on numerous data points, this device provides keratometry data separately for the anterior and posterior corneal surfaces. Pentacam's keratometry measurements are highly reliable and repeatable, even in corneas with pathological problems such as KCN^23^. Moreover, the user-friendliness of the device has made it widely used in screenings and epidemiological studies. Limited research has been conducted on the distribution of keratometry in the elderly population, with small sample sizes and insufficient focus on corneal astigmatism and detailed keratometry distribution. Additionally, the existing studies are limited by the lack of measurement using Pentacam.

According to the above, the present report aimed to investigate the distribution of keratometry and CA and their association with demographic factors, systemic parameters, anthropometric measures, ocular biometric indices, and refractive errors in an elderly population aged 60 years and above.

## Materials and methods

### Study design

The present report is part of a large population-based cross-sectional study (Tehran Geriatric Eye Study) that was conducted on elderly (aged 60 years and above) residents of Tehran City from January 2019 to January 2020.

The Tehran Geriatric Eye Study aimed to investigate visual impairment and ocular problems in the elderly population of Tehran. The sample size was calculated based on the prevalence of visual impairment as the primary outcome of interest. Considering a prevalence of 5.2% for visual impairment, precision of 1%, and confidence interval of 95%, the sample size was estimated at 1894 individuals. After applying a design effect of 1.5 and a non-response rate of 10% (sample size/(1-non-response rate)), the final sample size was calculated at 3155, which was rounded up to 3200 participants.

### Sampling

The study utilized the multi-stage stratified cluster sampling method. First, each of the 22 municipality districts of Tehran was considered as a stratum, and the population over 60 years in each district was obtained from the National Statistics Center. Next, a blocked map of each district was prepared and each block was defined as a cluster. One hundred and sixty clusters of 20 individuals were randomly selected from all 22 districts of Tehran and the number of clusters in each district was proportional to the population of that particular district. After identifying selected blocks, a sampling team visited their addresses and the first house of each block was considered as the cluster head. Subsequent households were selected through a counterclockwise movement. In this way, all elderly people (≥ 60 years) were invited to participate in the study and the sampling process continued until the required sample size in each cluster was completed. The objectives and steps of the study were fully explained to all invitees and they assured of the confidentiality of information. Once the samples were chosen, the examination date was set and the study participants were provided with transportation to the examination site at no cost. Upon arrival at the study site, a qualified individual gathered comprehensive demographic data from the participants.

### Ethical issues

All participants provided informed consent prior to their involvement in the study, adhering to the guidelines outlined in the Helsinki Declaration. The study protocol received approval from the Ethics Committee of the National Institute for Medical Research Development (NIMAD) under the Iranian Ministry of Health, with the ethics code IR.NIMAD.REC.1397.292.

### General examination

An individual was responsible for measuring anthropometric indices, starting with the height and weight of participants measured without shoes in standard conditions. Following this, systolic and diastolic blood pressures were measured twice with a ten-minute interval under standard conditions. If a significant difference was observed between the two measurements (≥ 10 mmHg in systolic pressure and/or ≥ 5 mmHg in diastolic pressure), a third measurement was conducted. The average of the first two measurements was recorded as the person's blood pressure, and if measured three times, the average of the two closest measurements was considered. Blood samples were also collected from each participant to evaluate blood sugar levels.

### Ocular examination

Ocular examination was performed in the next stage. Uncorrected visual acuity (UCVA), best-corrected visual acuity (BCVA), and presenting visual acuity (PVA) were tested using Smart LC 13, Medizs Inc. (Daejeon, South Korea) LED acuity chart at 6 m (m). The refraction was measured using ARK-510A, Nidek auto-refractometer/keratometer (Aichi, Japan). The anterior and posterior segment ocular examination was done by an ophthalmologist using B900, Haag-Streit slit-lamp biomicroscope (Bern, Switzerland) and a + 90 diopter (D) lens.

Corneal imaging and biometric measurements were eventually carried out using Oculus Pentacam AXL (Wetzlar, Germany). Biometric indices including flat (Kf) and steep (Ks) keratometry readings, CA, AL, central corneal thickness (CCT), ACD, and corneal diameter were obtained from Pentacam’s data.

### Exclusion criteria

Exclusion criteria were a history of any ocular surgery, a history of ocular trauma, corneal and anterior segment pathologies including pterygium, meibomian gland dysfunction (MGD), and corneal opacity. The outlier data was also excluded from the analysis, with outlier data being defined as exceeding 3 standard deviations (SD) from the mean. Additionally, images lacking an “OK” statement in the scan quality specification box or displaying an irregular map, and not showing any significant change after the instillation of artificial tears, were excluded from the report. Participants whose measured AL had a signal-to-noise (SNR) value of less than 6.3, were also excluded from the analysis.

### Quality control

An interview was carried out to gather demographic and ocular records by a qualified research assistant. The optometric examinations were conducted by two seasoned optometrists in a room with normal lighting conditions. The two examiners demonstrated high agreement in measuring uncorrected distance visual acuity (ICC: 0.994) and the spherical equivalent (SE) of subjective refraction (ICC: 0.967) among a pilot of 30 participants. Additionally, two trained optometrists conducted pilot Pentacam imaging on 30 subjects, which showed a high level of agreement in measuring the CCT(ICC: 0.971), keratometry (0.952).

To account for diurnal variations of ocular parameters, all participants were subjected to Pentacam imaging from 10 a.m. to 4 p.m. The necessary topographic and tomographic data was obtained from the Pentacam's axial maps. Examiners analyzed Pentacam images for substandard scanning quality and irregular maps potentially linked to tear film instability and dry eyes. In these cases, artificial tears were instilled and the imaging procedure was repeated after a 10-min interval.

Ocular data was inputted into a custom-designed data management program at the time of examinations. This program enabled the direct importation of data from the Pentacam’s software based on the patient identification code, eliminating the need for manual entry of data.

### Definitions

Refractive errors were defined based on the SE refraction. Participants with a SE worse than − 0.50 and + 0.50 D were considered myopic and hyperopic, respectively. The CA was calculated as a difference between Ks and Kf. To examine the axis of astigmatism, the axes of 30 to 150 (180 ± 30) degrees, 60 to 120 (90 ± 30) degrees, and the rest were defined as WTR, ATR, and oblique, respectively. The participants in this study were identified as having diabetes if their blood sugar level (BS) was higher than 200 mg/dl, their HbA1c level was over 6.4, they had a confirmed history of diabetes, or they were taking diabetes medication.

### Statistical analysis

This report analyzed the results of both eyes and used generalized estimating equation (GEE) analysis to control the effect of correlation of the fellow eyes. First, Kf, Ks, mean K, and CA were reported as mean, SD, and 95% confidence interval (CI) by age, sex, and refractive errors. To further describe these indices, 25%, 75%, 95%, and 99% percentiles as well as interquartile range (IQR) were reported in the studied population. The cluster sampling method was considered in calculating the standard error. The results were also standardized in terms of age and sex according to the population of Tehran using the direct method. Independent variables in this report were demographic factors (age and sex), systemic parameters (blood pressure and diabetes), anthropometric measures (height and body mass index), ocular biometric indices (AL, CCT, ACD, and corneal diameter), and refractive errors.

The simple and multiple GEE models were used to examine the relationship between mean K and CA with independent variables, and the model coefficients were reported along with 95% CIs. The initial analysis involved examining the relationship between each variable with keratometry and CA, using a simple model. Subsequently, a multiple model was constructed, encompassing all independent variables. After evaluating collinearity and interactions among the variables, the final model was presented including all variables that were found to be statistically significant (*p* value < 0.05).

### Ethics approval and consent to participate

Informed consent was obtained from all participants. The principles of the Helsinki Declaration were followed in all stages of this study. The protocol of the study was approved by the Ethics Committee of the National Institute for Medical Research Development (NIMAD) under the auspices of the Iranian Ministry of Health.

## Results

Tehran Geriatric Eye study invited 3791 people aged 60 years and above from Tehran, the capital of Iran. Of these, 3310 individuals participated in the study. After applying the exclusion criteria, 4244 eyes from 2268 participants were analyzed for this report. The mean age of the participants analyzed was 66.9 ± 5.6 (range: 60 to 95) years, and 1332 (58.7%) of them were female. The mean spherical equivalent was 0.39 ± 1.81 D.

**Fig. 1:**
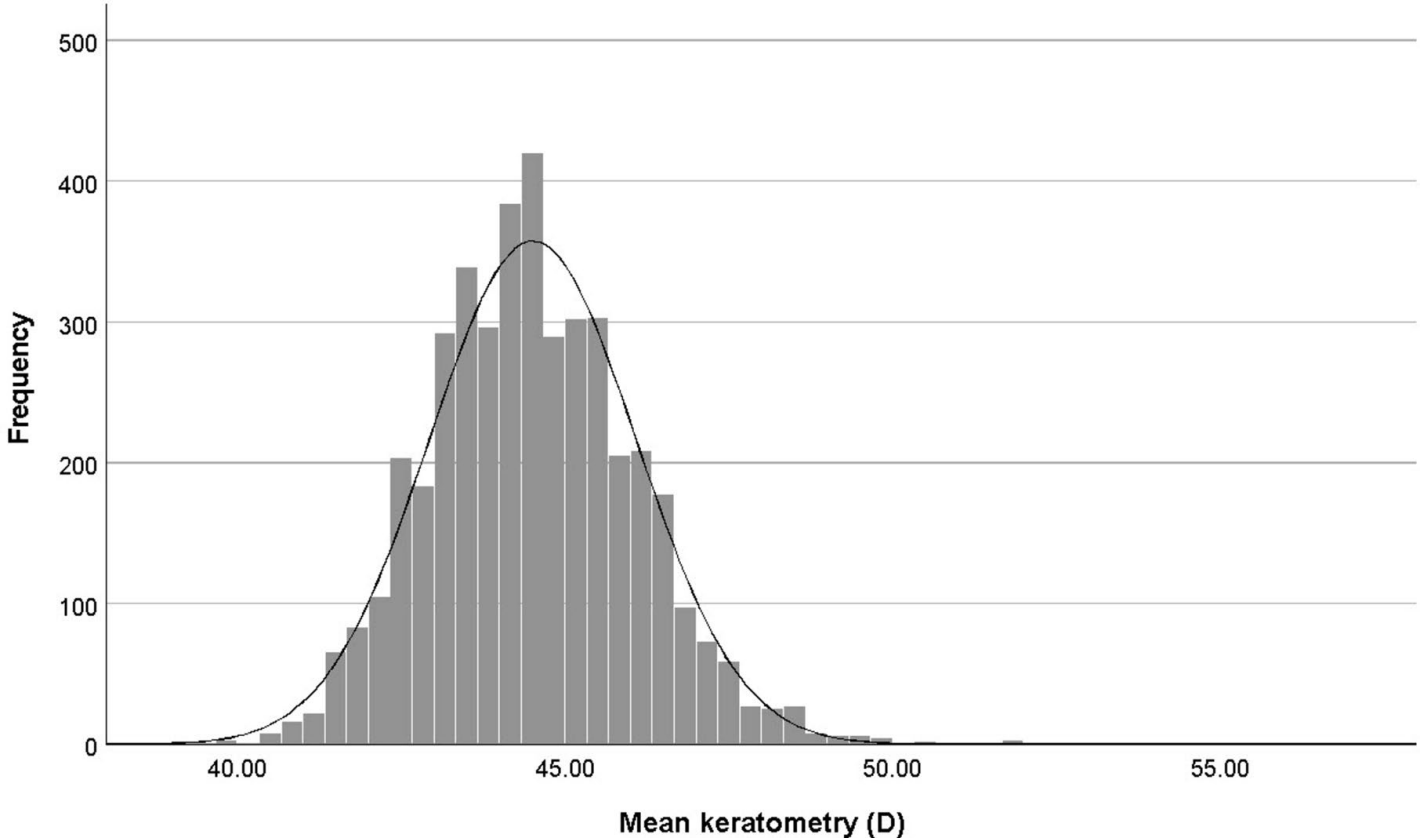
The distribution of mean keratometry in an elderly population.

Table 1 presents the average, SD, and 95% CI of Kf, Ks, mean K, and CA in the whole sample and by age, sex, and refractive errors. The average, SD, and 95% CI of Kf, Ks, mean K and CA were 44.02 ± 1.58 D (95% CI 43.94–44.1), 44.86 ± 1.67 D (95% CI 44.78–44.94), 44.44 ± 1.58 D (95% CI 44.36–44.52), and 0.84 ± 0.74 D (95% CI 0.81–0.87), respectively. Figure 1 illustrates the distribution of these variables. The skewness of these variables was 0.261, 0.691, 0.476, and 3.00, respectively and their kurtosis was 0.730, 3.398, 1.245, and 15.327, respectively.

**Table 1:** Flat keratometry, steep keratometry, mean keratometry (mean-K), corneal astigmatism by age, gender and refractive errors.

	Flat keratometry	Steep keratometry	Mean-k	Corneal astigmatism
Total	44.02 ± 1.58 (43.94; 44.1)	44.86 ± 1.67 (44.78; 44.94)	44.44 ± 1.58 (44.36; 44.52)	0.84 ± 0.74 (0.81; 0.87)
Gender				
Male	43.72 ± 1.37 (43.62; 43.83)	44.57 ± 1.45 (44.46; 44.68)	44.15 ± 1.36 (44.04; 44.25)	0.84 ± 0.68 (0.8; 0.89)
Female	44.33 ± 1.73 (44.23; 44.44)	45.17 ± 1.83 (45.05; 45.28)	44.75 ± 1.74 (44.64; 44.85)	0.83 ± 0.78 (0.8; 0.87)
Age				
60–64	43.9 ± 1.56 (43.78; 44.02)	44.74 ± 1.62 (44.62; 44.87)	44.32 ± 1.54 (44.2; 44.44)	0.84 ± 0.75 (0.8; 0.89)
65–69	44.06 ± 1.68 (43.94; 44.18)	44.87 ± 1.83 (44.75; 45)	44.46 ± 1.71 (44.35; 44.58)	0.81 ± 0.77 (0.77; 0.86)
70–74	44.19 ± 1.59 (44.03; 44.35)	45.02 ± 1.64 (44.85; 45.19)	44.6 ± 1.58 (44.44; 44.77)	0.83 ± 0.69 (0.77; 0.88)
75–79	44.21 ± 1.36 (43.95; 44.47)	45.03 ± 1.45 (44.73; 45.34)	44.62 ± 1.38 (44.34; 44.9)	0.82 ± 0.57 (0.73; 0.92)
≥ 80	44.06 ± 1.33 (43.68; 44.43)	45.07 ± 1.39 (44.7; 45.44)	44.56 ± 1.31 (44.2; 44.92)	1.01 ± 0.72 (0.82; 1.21)
Refractive errors				
Emmetropia	44.08 ± 1.53 (43.95; 44.2)	44.89 ± 1.59 (44.78; 45.01)	44.49 ± 1.52 (44.37; 44.61)	0.82 ± 0.72 (0.77; 0.86)
Myopia	44.25 ± 1.71 (44.1; 44.4)	45.39 ± 1.87 (45.21; 45.56)	44.82 ± 1.72 (44.66; 44.98)	1.14 ± 0.98 (1.05; 1.22)
Hyperopia	43.91 ± 1.55 (43.81; 44.02)	44.65 ± 1.59 (44.54; 44.75)	44.28 ± 1.54 (44.18; 44.38)	0.74 ± 0.59 (0.71; 0.77)

Table 2 shows the 25%, 75%, 95%, and 99% percentiles as well as IQR of Kf, Ks, mean K, and CA in the studied eyes by age, sex, and refractive errors. The 95% and 99% percentiles of the mean K were 47.1 and 48.6 D, respectively. The 95% and 99% percentiles of CA were 1.2 and 3.5 D, respectively.

**Table 2:** The percentiles and interquartile range (IQR) of Flat keratometry, Steep keratometry, mean keratometry (mean-K) and corneal astigmatism by age, gender and refractive errors.

	25%	75%	95%	99%	IQR	25%	75%	95%	99%	IQR	25%	75%	95%	99%	IQR	25%	75%	95%	99%	IQR
Parameter	Flat keratometry					Steep keratometry					Mean-K					Corneal astigmatism				
All	43	45.1	46.7	48	2.1	43.8	45.9	47.6	49.5	2.1	43.4	45.5	47.1	48.6	2.1	0.4	1.1	2.1	3.5	0.7
Gender																				
Male	42.7	44.7	46.3	47.7	2	43.5	45.6	47.3	49	2.1	43.2	45.2	46.7	48.2	2	0.4	1	2.2	3.9	0.6
Female	43.2	45.3	46.9	48.2	2.1	44	46.2	47.8	49.6	2.2	43.7	45.8	47.3	48.6	2.1	0.4	1.1	2	3.5	0.7
Age (year)																				
60–64	42.9	45.1	46.5	48.2	2.2	43.7	45.8	47.5	49.7	2.1	43.4	45.5	47	48.7	2.1	0.4	1.1	2.2	3.5	0.7
65–69	43.1	45.2	46.6	47.9	2.1	43.8	46	47.7	49.3	2.2	43.4	45.6	47.1	48.6	2.2	0.4	1	1.9	3.7	0.6
70–74	43.2	45.2	47.1	48.1	2	43.9	46	47.8	49.3	2.1	43.6	45.7	47.4	48.6	2.1	0.4	1	2.1	3.5	0.6
75–79	43.1	45.2	46.8	47.6	2.1	43.8	46	47.9	49	2.2	43.4	45.6	47.4	48.2	2.2	0.4	1	1.8	3.3	0.6
≥ 80	42.9	44.8	46.5	47.6	1.9	44	45.9	47.6	50.1	1.8	43.5	45.3	47	48.5	1.8	0.5	1.3	3.3	4.5	0.8
Refractive errors																				
Emmetropia	43.1	45.2	46.6	47.8	2.1	43.9	46	47.5	49.3	2.1	43.5	45.6	47.1	48.5	2.1	0.4	1	2.1	3.4	0.6
Myopia	43.2	45.4	47.2	48.9	2.2	44.3	46.5	48.5	51.2	2.2	43.8	46	47.8	49.8	2.2	0.5	1.5	3	5.4	1
Hyperopia	42.9	45	46.5	47.7	2.1	43.6	45.7	47.3	48.8	2.1	43.3	45.4	46.9	48.2	2.1	0.4	1	1.7	3.1	0.6

**Fig. 2:**
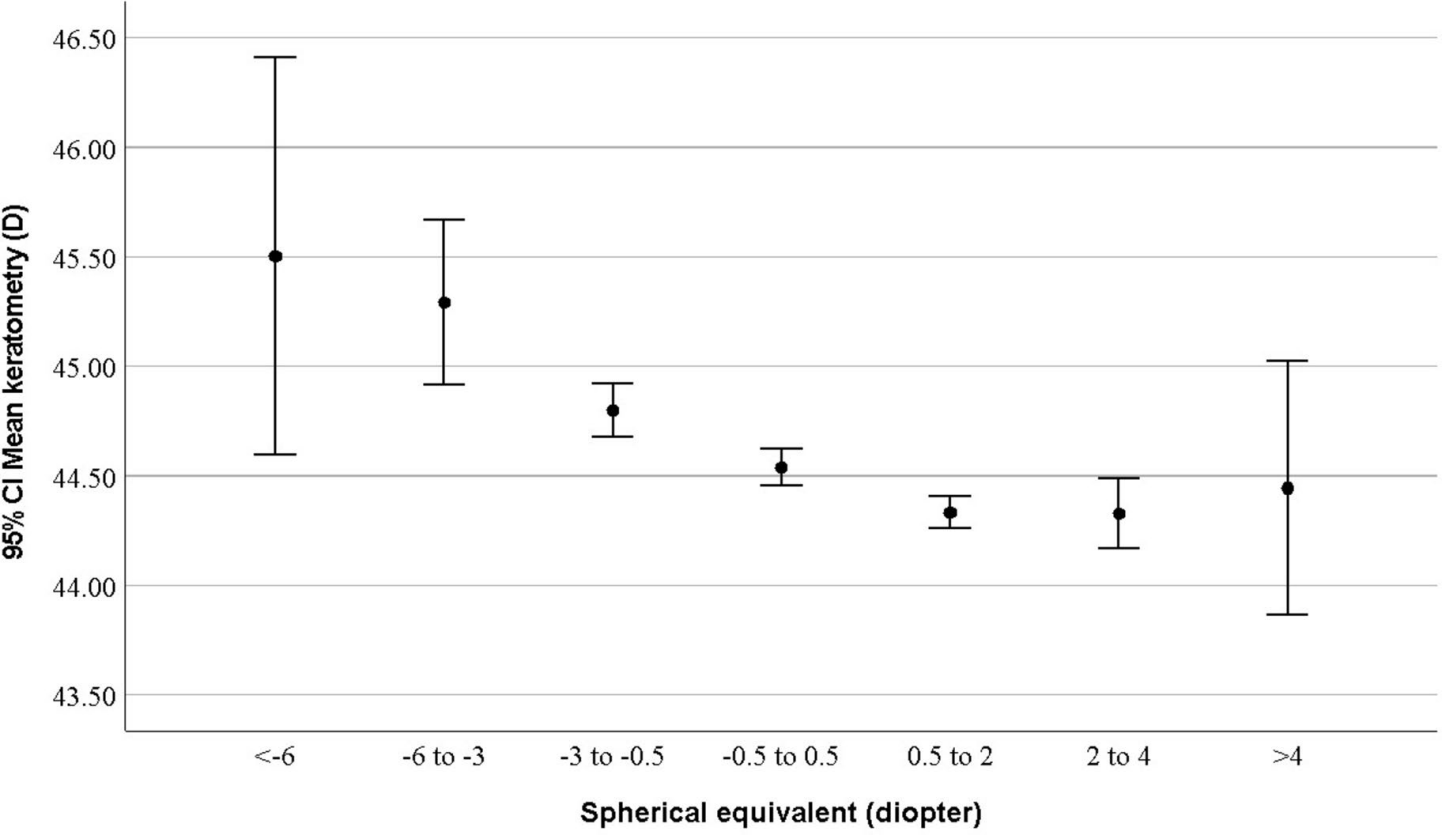
The distribution of mean keratometry according to severity of refractive errors.

As seen in Table 1, the mean K was significantly higher in women compared to men (*p* < 0.001). Changes in the mean K with age were not statistically significant. The mean K was the highest in myopes and the lowest in hyperopes (*p* < 0.001). As seen in Fig. 2, the highest mean K was observed in participants with myopia worse than − 6.00 D.

The CA was not significantly different between males and females (*p* = 0.644). The mean CA did not show significant changes up to 80 years of age; however, it significantly increased thereafter. The average CA was the highest and lowest in myopes and hyperopes, respectively.

The relationship between mean K with demographic, biometric, and corneal indices was examined through simple and multiple GEE models. The results of these models are shown in Table 3. According to the final GEE model, the mean K was significantly lower in females than in males. Moreover, the mean K was significantly higher in myopes and lower in hyperopes. The multiple GEE model showed that the mean K was inversely related to the AL, height, ACD, corneal diameter, and CCT. There was also a statistically significant direct association between mean K and systolic blood pressure.

**Table 3:** The association between average keratometry and study variables by simple and multiple generalized estimating equation (GEE).

Study variables	Coefficient (95% CI)	*p* value	Coefficient (95% CI)	*p* value
Female gender	0.531 (0.403; 0.659)	< 0.001	− 0.145 (− 0.269; − 0.021)	0.022
Age				
60–64	0	–		NR
65–69	0.103 (− 0.048; 0.254)	0.182		
70–74	0.181 (− 0.002; 0.365)	0.053		
75–79	0.091 (− 0.179; 0.361)	0.509		
≥ 80	0.112 (− 0.248; 0.472)	0.543		
Refractive errors				
Emmetropia	0			
Myopia	0.249 (0.172; 0.326)	< 0.001	0.464 (0.389; 0.538)	< 0.001
Hyperopia	− 0.165 (− 0.227; − 0.103)	< 0.001	− 0.318 (− 0.377; − 0.259)	< 0.001
Education	− 0.035 (− 0.047; − 0.023)	< 0.001		NR
Diabetes	0.104 (− 0.041; 0.248)	0.159		NR
Systolic blood pressure	0.006 (0.003; 0.009)	< 0.001	0.003 (0.001; 0.005)	0.004
Body mass index	0.007 (− 0.007; 0.021)	0.335		NR
Height	− 0.045 (− 0.051; − 0.038)	< 0.001	− 0.023 (− 0.03; − 0.016)	< 0.001
Axial length	− 0.751 (− 0.798; − 0.703)	< 0.001	− 0.919 (− 0.968; − 0.871)	< 0.001
Anterior chamber depth	− 0.249 (− 0.407; − 0.09)	0.002	0.988 (0.858; 1.117)	< 0.001
Central corneal thickness	− 0.003 (− 0.005; − 0.002)	< 0.001	− 0.005 (− 0.006; − 0.004)	< 0.001
Corneal diameter	− 0.889 (− 0.977; − 0.801)	< 0.001	− 0.844 (− 0.926; − 0.761)	< 0.001

NR: not retained.

Multiple model fit: AIC = 11,796.6; BIC = 11,859.89.

Examining the relationship of CA with the studied variables using the multiple GEE model (Table 4) showed a statistically significant direct relationship between CA with myopia, mean K, and AL. In addition, the CA was significantly inversely related to ACD and CCT.

**Table 4:** The association between corneal astigmatism and study variables by simple and multiple generalized estimating equation (GEE).

Study variables	Coefficient (95%CI)	*p* value	Coefficient (95%CI)	*p* value
Female gender	− 0.013 (− 0.067; 0.041)	0.644		NR
Age				
60–64	0			NR
65–69	− 0.039 (− 0.102; 0.024)	0.223		
70–74	− 0.031 (− 0.107; 0.046)	0.435		
75–79	− 0.025 (− 0.138; 0.089)	0.671		
≥ 80	0.19 (0.036; 0.344)	0.016		
Refractive errors				
Emmetropia	0			NR
Myopia	0.267 (0.203; 0.331)	< 0.001	0.197 (0.131; 0.263)	< 0.001
Hyperopia	− 0.086 (− 0.137; − 0.035)	0.001	− 0.027 (− 0.08; 0.025)	0.308
Education	− 0.001 (− 0.006; 0.004)	0.807		NR
Diabetes	0.003 (− 0.057; 0.064)	0.911		NR
Systolic blood pressure	0.002 (0; 0.003)	0.015		NR
Height	− 0.002 (− 0.005; 0.001)	0.179		NR
Body mass index	0.001 (− 0.005; 0.006)	0.83		NR
Mean-k	0.059 (0.043; 0.076)	< 0.001	0.083 (0.061; 0.104)	< 0.001
Axial length	0.035 (0.006; 0.064)	0.017	0.127 (0.084; 0.17)	< 0.001
Anterior chamber depth	− 0.056 (− 0.136; 0.023)	0.165	− 0.209 (− 0.297; − 0.12)	< 0.001
Central corneal thickness	− 0.003 (− 0.003; − 0.002)	< 0.001	− 0.002 (− 0.003; − 0.001)	< 0.001
Corneal diameter	− 0.076 (− 0.137; − 0.016)	0.014		

NR: not retained.

Multiple model fit: AIC = 8815.388; BIC = 8859.702.

The prevalence of various types of CA based on a cut-off > 0.50 D was as follows; WTR: 32.5% (95% CI 30.6–34.4), ATR: 18.2% (95% CI 16.7–19.7), and oblique: 10.0% (95% CI 9.1–11.0).

**Fig. 3:**
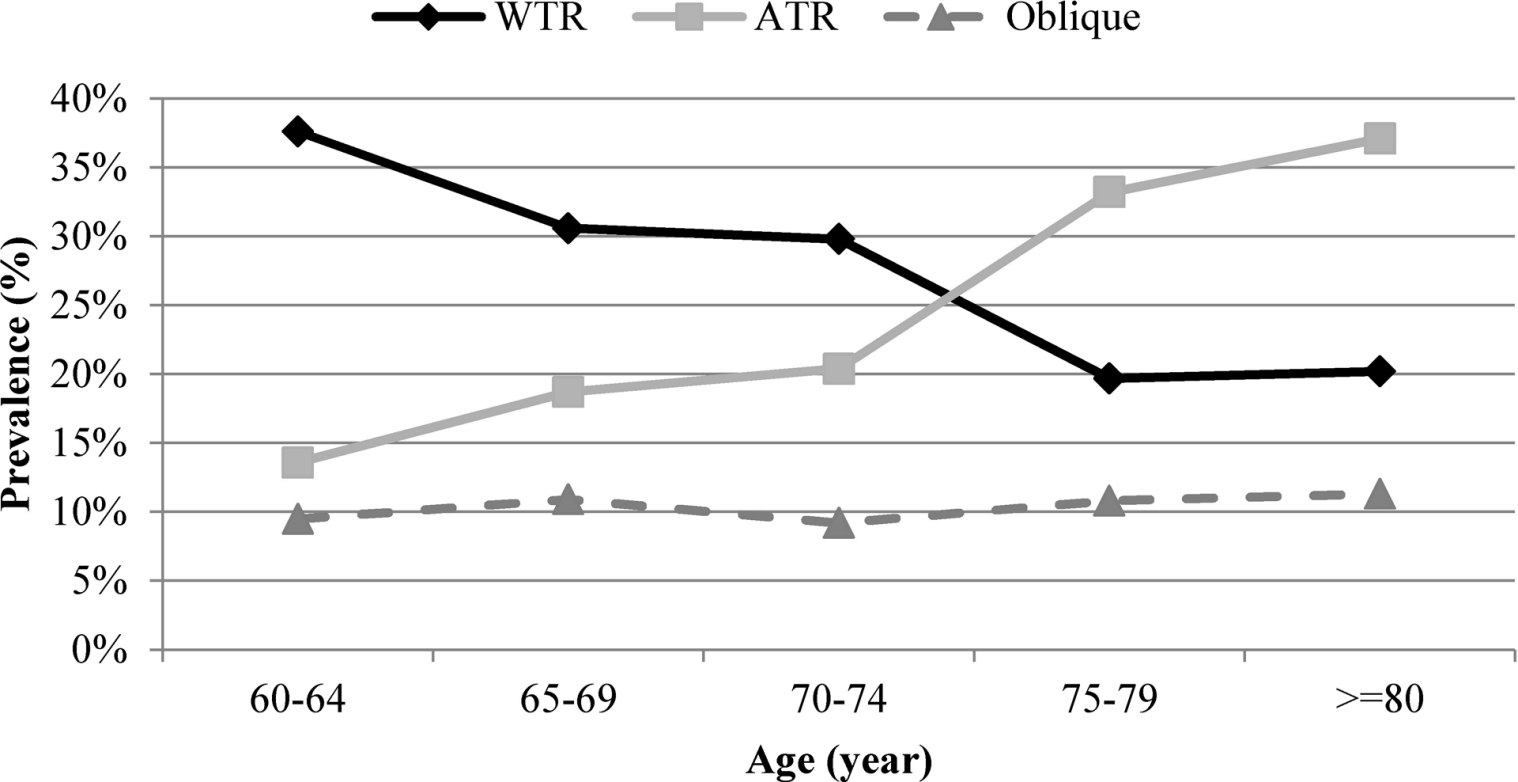
The prevalence of the different types of corneal astigmatism (with-the-rule: WTR; against-the-rule: ATR; Oblique) by age.

Our findings showed that after adjusting for age in GEE model, WTR astigmatism was significantly more prevalent in men while ATR astigmatism was significantly more prevalent in women (*p* < 0.001). There was no statistically significant relationship between oblique astigmatism and sex (*p* = 0.631). Figure 3 shows the age-related changes in CA in participants with CA greater than 0.5 D. As seen in Fig. 3, the prevalence of WTR astigmatism decreased with advancing age from 37.6% in the age group 60–64 to 20.2% in the age group 80 years and above. The prevalence of ATR astigmatism showed an increasing pattern from 13.0% in participants aged 60 to 64 years to 37.1% in those aged 80 years and above (*p* < 0.001). The changes in oblique astigmatism with age were not statistically significant (*p* = 0.601).

## Discussion

This report presented the distribution of keratometry and CA among individuals aged 60 years and above, along with their association with demographic factors, systemic parameters, anthropometric measures, various ocular biometric indices, and refractive errors.

Although similar studies have examined keratometry, CA, and their relationships in this age group, the present report evaluated these associations using a larger sample size and taking into account more indicators such as biometric components including AL, CCT, ACD, and corneal diameter^1,24–27^. Given different age distributions and the use of various instruments for keratometric measurements, our results should be compared with other studies with caution. According to the findings of the present study, the mean Kf and Ks were 44.02 and 44.86 D, respectively. A study on an Iranian population aged 6–90 years showed that the average Kf and Ks were 42.98 and 43.98 D, respectively^1^. Another study conducted in Iran (Shahroud City) reported mean Kf and Ks values of 43.27 and 44.19 D, respectively, in subjects aged 40–64 years^28^. A study on a Chinese population over 40 years using swept-source optical biometry found an average mean K of 44.26 D^29^. A review of the literature in this regard shows that the mean K in our population is not much different from previous studies. In general, studies with an older average age reported higher values of mean K^1,24–29^. The measuring device may also be involved in slight discrepancies among different studies.

Our study did not show a statistically significant relationship between mean K and age. There are conflicting reports regarding the association between keratometry and age^1,28,30,31^. Some previous studies found that the mean K increases with age; these studies mostly had a wide age range from childhood to old age^1,32^. The Shahroud Eye Cohort Study showed a slight increase in the mean K between the ages of 40 and 64 years^30^. An overall overview of keratometry changes with age reveals a U-shaped pattern. Keratometry is high under 7 years and decreases with age to maintain the emmetropization phenomenon^33^. Then, it increases again slightly and is almost constant after 60 years.

In the present study, the mean K was higher in women compared to men based on the simple GEE model. However, the multiple GEE model showed a significantly lower mean K in women by controlling the effect of other variables. Most previous studies found a higher mean K in women compared to men^1,24,30,34–37^. A study on Chinese students reported a significantly higher radius of corneal curvature in boys than in girls, or in other words, lower keratometry in boys than in girls^38^. A study on children and middle-aged people in Shahroud (Iran) also reported higher keratometry in females compared to males^30^. The difference in the result of the present study with other studies may result from age and racial differences. Moreover, we examined the relationship between keratometry and sex using the multiple GEE model after removing the effects of other variables. This is while most previous studies examined this relationship using simple regression models. So, the confounders, especially the AL, seem to play a significant role in this relationship. Considering hormonal and physiological differences, there is expected to be a difference between men and women in keratometry.

As observed in the final GEE model (Table 3), the mean K was higher in myopes and lower in hyperopes than in emmetropes. In addition, the mean k was significantly inversely related to the AL, height, and ACD. We believe that these relationships should be analyzed from different viewpoints. Steeper keratometry in myopia has been reported in previous studies^1,31,39,40^, and it makes sense for an individual to be myopic due to high corneal power. On the other hand, the mean K is directly related to CA, and part of this relationship probably originates from defining myopia based on the SE refraction. Previous studies have shown that the AL and the corneal power are inversely related to maintain the emmetropization process^33^. So, the indirect association between mean K and AL is expected, and our study confirmed this relationship in elderly people. Although the observed relationship may originally relate to a young age, part of the relationship may have been diminished to maintain emmetropization due to lens changes in old age. The direct correlation of ACD and height with the AL is also a reason for their inverse relationships with keratometry, confirmed by previous studies. The inverse association between the corneal diameter and corneal thickness with keratometry has also been observed in previous studies^41^. It seems that as the corneal diameter increases, the cornea tends to become flatter and thinner. This finding is important in refractive surgery candidates and KCN patients.

The average CA in the present study was 0.84 D and about 25% of participants had a CA of 1.00 D and above. According to percentiles, 5% and 1% of the study participants had a CA of more than 1.2 and 3.5 D, respectively. A look at previous studies and comparison with our results indicates that CA increases in older ages and the cornea tends to become more irregular with aging^3–5,25,29,42^. Although, these cases should be checked for KCN, increased CA with age is also anticipated in normal corneas due to age-related changes in corneal hydration, increased measurement errors, and dry eyes, and this finding should be considered in cataract surgery and even IOL power calculation.

After adjusting for age, the WTR astigmatism was more prevalent in men while the ATR astigmatism was more prevalent in women. The relationship between various types of astigmatism and sex has already been investigated; however, previous studies have mainly addressed refractive astigmatism^43–46^. Since the CA is a major component of refractive astigmatism, the CA appears to follow the pattern of refractive astigmatism. The results of previous studies in this regard are contradictory. In the Shahroud Eye study, oblique astigmatism was reported to be more common in women than in men^45^. Mandel etal. showed that females have a higher prevalence of WTR astigmatism^43^. Huynh et al.^44^ reported a higher prevalence of WTR astigmatism in girls and a higher prevalence of oblique astigmatism in boys. It should be noted that Mandel and Huynh’s studies were conducted in the young and adolescent age groups and it is difficult to compare the results given the age-related changes in the axis of astigmatism^43,44^. Aydin et al.^47^ found significant changes in the astigmatism axis before and after menopause in women. So, this issue should be considered given the age group of the present study. Although this relationship is difficult to explain, sex-related differences in palpebral fissure slant can also play a role. The present study found a shift from WTR to ATR astigmatism with age. This finding has already been proven from cross-sectional and cohort studies^1,4,37,42,44–46^. In general, most studies have proposed the effect of eyelid pressure on corneal curvature^20^. Eyelid pressure is higher at a young age due to the tonus of the eyelid muscles, resulting in WTR astigmatism. As the eyelid muscles become weaker with age, the eyelid pressure decreases and the CA axis shifts toward the ATR direction.

The correlation between sex and ocular biometric parameters has led to sex being recognized as a significant factor in the development of newer formulas for calculating IOL power^48^. This inclusion of sex has been found to enhance the precision of these newer IOL power calculation methods^49^.

The refractive error of the eye is determined by the biometric parameters, and research has shown that sex can also influence the refractive errors^50^. Several studies have indicated that men tend to have a higher prevalence of myopia compared to women^51^. This difference may be attributed to the fact that men generally have longer ALs than women^52^.

The present study has both strengths and limitations that warrant attention. The study's strengths include a large sample size, robust sampling method tailored to each region's population, and efforts to minimize selection bias, and representativeness of the general population. However, we also made efforts to mitigate potential sources of error. We minimized the potential impact of dry eyes by conducting measurements after applying artificial tears, and we reduced daily fluctuations in ocular indices by limiting imaging time. Additionally, to ensure the reliability and validity of the results, we provided training to the examiners and assessed their agreement.

## Data Availability

The datasets used and/or analyzed during the current study available from the corresponding author on reasonable request.
